# Neurobiology of Dystonia: Review of Genetics, Animal Models, and Neuroimaging

**DOI:** 10.3390/brainsci15070767

**Published:** 2025-07-19

**Authors:** Jamir Pitton Rissardo, Andrew McGarry, Yiwen Shi, Ana Leticia Fornari Caprara, Ian M. Walker

**Affiliations:** 1 Neurology Department, Cooper University Hospital, Camden, NJ 08103, USAfornari-caprara-ana@cooperhealth.edu (A.L.F.C.);; 2 Cooper Medical School, Rowan University, Camden, NJ 08103, USA

**Keywords:** pathophysiology, neurobiology, mechanism, torsion, muscle contraction, dystonia

## Abstract

Over the past decade, substantial progress has been made in understanding the pathophysiology of dystonia. The number of identified genes has surged—exceeding 400 by 2024—with approximately 76.6% linked to neurodevelopmental disorders. Despite this, the genetic diagnostic yield remains modest (12–36%), and many newly discovered genes have yet to reveal novel mechanistic insights. The limited number of studies exploring dystonia-related pathways in animal models restricts the generalizability of findings to human disease, raising concerns about their external validity. Developing experimental models remains a challenge, particularly given the importance of critical developmental windows—periods during central nervous system maturation when disruptions can have lasting effects. Some models also exhibit delayed symptom onset, prompting a shift toward faster-developing organisms such as Drosophila. There is a pressing need for standardized, scalable protocols that enable precise evaluation of specific neural tissues. Advances in neuroimaging have improved our understanding of dystonia-related brain networks at both regional and whole-brain levels. The emerging concept of “network kernels” has provided new perspectives on brain connectivity. However, future imaging studies should incorporate effective connectivity analyses to distinguish between hemodynamic and neuronal contributions and to clarify neurobiological pathways. This review synthesizes current knowledge from genetics, animal models, and neuroimaging to present an integrated view of dystonia’s neurobiological underpinnings.

## 1. Introduction

Dystonia was once considered a psychiatric disorder of unknown origin. In 1911, Dr. Hermann Oppenheim described the sustained muscle spasms of “dystonia musculorum deformans,” formally recognizing it as a neurological condition [1]. A century later, advances in genetics began to uncover the molecular basis of dystonia, leading to the development of animal models. However, many of these models rely on non-specific lesion-based approaches that aim to replicate dystonic phenotypes rather than model specific disease mechanisms, raising concerns about replicability and clinical relevance. The identification of critical developmental windows—periods during central nervous system (CNS) maturation when disruptions can cause lasting functional impairments—has been pivotal in refining experimental protocols. These windows are now recognized as key to understanding the motor dysfunction observed in dystonia. In parallel, neuroimaging technologies have evolved, enabling the exploration of hemodynamic and functional brain changes with increasing precision. This review integrates current insights from genetics, animal models, and neuroimaging to provide a comprehensive overview of the neurobiology of dystonia.

### Search Strategy

To ensure a comprehensive and focused review, we included studies published between 2010 and 2025 that investigated the genetic, neuroimaging, and animal model aspects of dystonia. The inclusion criteria were (1) original research articles or systematic reviews; (2) studies involving next-generation sequencing (NGS), including whole-exome and whole-genome sequencing; (3) neuroimaging studies using modalities such as fMRI, PET, or DTI in dystonia patients; and (4) animal models specifically designed to investigate dystonia pathophysiology. Exclusion criteria included case reports, non-peer-reviewed articles, and studies not available in English. Literature was identified through PubMed, Scopus, and Web of Science using keywords such as “dystonia,” “genetics,” “NGS,” “neuroimaging,” “animal models,” and “pathophysiology.” Reference lists of included articles were also screened for additional relevant studies.

## 2. Insights from Genetics

Dr. Hermann Oppenheim first introduced the term dystonia in 1911 [1]. However, the genetic basis of the disorder remained elusive until 1997, when the TOR1A (DYT1) gene was identified as the cause of early-onset isolated dystonia. Over the following two decades, progress in identifying additional dystonia-related genes was slow, largely due to the limitations of first-generation sequencing technologies, which relied on targeted single-gene testing. This changed in 2010 with the advent of next-generation sequencing (NGS), including short-read panel sequencing, whole-exome sequencing (WES), and whole-genome sequencing (WGS), as well as more recent long-read genome sequencing. These advances have significantly expanded the catalog of dystonia-associated genes (Table S1).

### 2.1. Genetic Classification

There are multiple classification systems for dystonia described in the literature. The Online Mendelian Inheritance in Man (OMIM^®^) database categorizes dystonia phenotypes numerically, ranging from DYT1 to DYT37 (Table S2). In contrast, the Movement Disorder Society Genetic Mutation Database (MDSGene) uses a classification based on clinical symptomatology and phenomenology (Table S3) [2]. Traditional classification systems, which relied heavily on phenotypic presentation, have largely been phased out due to significant overlap between clinical features. The current standard favors a gene-based nomenclature, using the “DYT” prefix followed by the specific gene name—for example, “DYT-TOR1A.” This updated approach offers greater precision and consistency and is now widely adopted in both clinical practice and research settings.

### 2.2. Genetics in Dystonia

Next-generation sequencing (NGS) has emerged as a powerful tool for identifying genetic variants associated with dystonia across diverse populations. Most population-based studies to date have utilized whole-exome sequencing (WES), with only one study employing whole-genome sequencing (WGS) (Table 1). Reported diagnostic yields range from 12% to 36%, indicating that a substantial proportion of individuals (64% to 88%) remain genetically undiagnosed. A key limitation of these studies is their predominant focus on generalized dystonia, often without distinguishing between focal and generalized forms in terms of outcomes or prognostic implications.

The type of NGS technology used plays a significant role in the diagnosis of genetic dystonia. Whole-exome sequencing (WES), while widely used, is limited to detecting mutations within coding regions and may miss short repeat expansions or structural variants, particularly when breakpoints occur at exon boundaries [12]. In contrast, whole-genome sequencing (WGS) offers broader coverage, including non-coding regions, and is better suited for identifying complex genomic alterations. Long-read sequencing technologies further enhance detection capabilities by resolving repetitive regions and structural variants with greater accuracy. However, these methods are often constrained by higher costs and longer processing times. An alternative strategy to improve diagnostic yield—especially in patients with severe phenotypes (clinical score ≥ 3)—is to reanalyze existing genome sequencing datasets. This approach has been shown to increase diagnostic rates by up to 7% [13].

A recent study implemented whole-genome sequencing (WGS) in individuals with dystonia who remained undiagnosed after prior genetic testing, aiming to identify previously undetected disease-associated variants. The cohort included 564 patients with a strong clinical suspicion of monogenic dystonia but negative exome sequencing results. WGS increased the diagnostic yield by an additional 10%, uncovering variants that were missed by exome analysis. These included mitochondrial DNA mutations, repeat expansions, copy number variants, structural variants, and mutations in non-covered coding regions, intronic regions, and RNA genes. Notable examples include cases of KMT2B-related dystonia caused by a coding mutation not captured by WES [14] and BCL11B-related dystonia resulting from copy number variants that were difficult to detect using standard exome techniques [15].

An important observation is the directly proportional relationship between the number of identified dystonia-related genes and the size of the study population. For instance, in 2019, approximately 90 genes were identified in a cohort of 708 individuals, whereas by 2024, this number had increased to 210 genes in a sample of 1825 participants [16]. This trend highlights how larger cohorts enhance the power of genetic discovery. The identification of these genes offers several clinical advantages, including personalized genetic counseling for affected families, the ability to map converging molecular pathways, and the development of targeted, gene-specific therapeutic strategies.

In the current database of dystonia-associated genes, approximately 76.6% are linked to neurodevelopmental conditions (Table S4), while only 23.4% are associated with degenerative diseases, mixed pathologies, or mechanisms that remain unclear [17]. This strong neurodevelopmental association is supported by the observation that several dystonia subtypes—such as pediatric complex dystonia, early-onset isolated dystonia, dystonia-parkinsonism, and late-onset focal dystonia—are often linked to lesions in the developing brain [18]. Early studies also reported delayed-onset dystonia in individuals with static encephalopathy [19] and in those affected by perinatal or early childhood asphyxia [20]. A large-scale study of individuals with neurodevelopmental disorders, primarily of genetic or monogenic origin, found that approximately 10% of the participants exhibited dystonia, and nearly 3% had ataxia [21]. Neurodevelopmental disorders can manifest a broad spectrum of neuropsychiatric phenotypes, including dystonia, often through mechanisms such as impaired synaptic transmission and disrupted calcium homeostasis [22]. Moreover, there appears to be a mechanistic link between genes involved in nervous system development and those implicated in dystonia. For example, modulation of the neurodevelopmental gene RANBP17—a key regulator of nucleocytoplasmic transport—has been associated with the expression of DYT1, a gene characteristic of isolated dystonia [23].

### 2.3. Converging Genes and Causative Pathways

**Figure 1:**
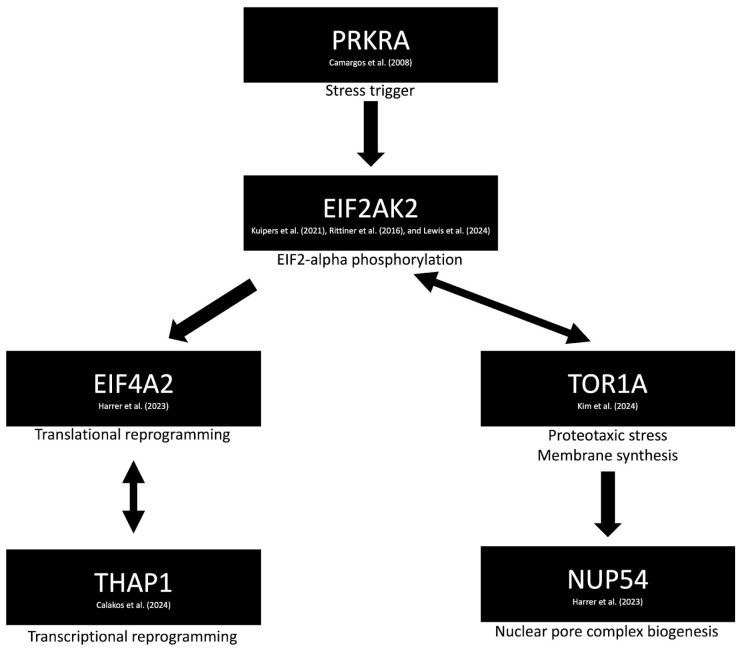
Proposed molecular mechanism of dystonia involving impaired autophagy (via EIF2AK2 [24,25,26] and EIF4A2 [27]) and dysregulation of the integrated stress response pathway (via PRKRA [29]). TOR1A is implicated in the maturation and modulation of the neuronal nuclear pore complex [31], particularly through its interaction with NUP54 [28]. Additional genes, such as THAP1, are involved in transcriptional reprogramming, further contributing to the pathophysiological cascade [30].

The identification of a diverse array of dystonia-related genes has provided valuable insights into potential causative molecular pathways. One such pathway involves a network of genes including EIF2AK2 [24,25,26], EIF4A2 [27], NUP54 [28], PRKRA [29], THAP1 [30], and TOR1A [31], as revealed through the STRING (Search Tool for the Retrieval of Interacting Genes/Proteins) version 12 database [32] (Figure 1) (Table S5). Notably, TOR1A has been linked to the maturation of the neuronal nuclear pore complex (NPC), a process that is upregulated during neurodevelopment. Disruption of NPC biogenesis has been implicated in the pathogenesis of dystonia [31]. Additionally, mutations in NUP54—classified as DYT-NUP54 (DYT37)—have been associated with isolated dystonia accompanied by striatal lesions, highlighting a potential neuroimaging biomarker that warrants further investigation [28].

**Figure 2:**
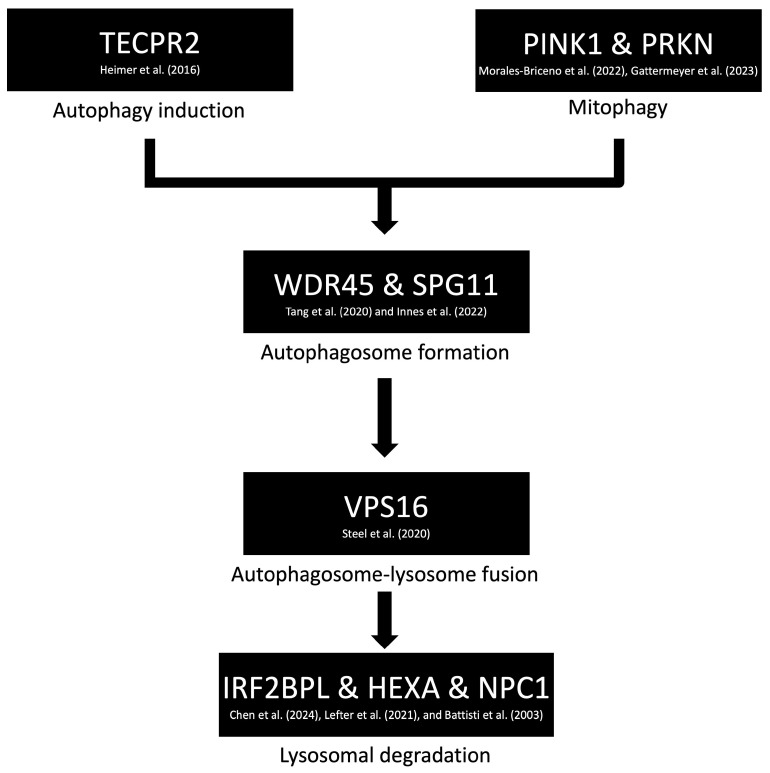
Proposed molecular mechanism of dystonia involving disruptions in autophagy and lysosomal pathways. Key genes implicated in this pathway—such as HEXA [33], IRF2BPL [34], NPC1 [35], PINK1 [36], PRKN [37], SPG11 [38], TECPR2 [39], VPS16 [40], and WDR45 [41]—are associated with impaired degradation and recycling of cellular components. These dysfunctions may contribute to neurodegenerative processes in which dystonia appears as part of a broader clinical phenotype.

Another pathway implicated in dystonia involves impaired autophagy and lysosomal dysfunction. Genes associated with this mechanism include HEXA [33], IRF2BPL [34], NPC1 [35], PINK1 [36], PRKN [37], SPG11 [38], TECPR2 [39], VPS16 [40], and WDR45 [41], as identified through the STRING version 12 database (Figure 2) (Table S5). Notably, a similar autophagy–lysosomal pathway has been extensively studied in Parkinson’s disease, suggesting potential overlap in the underlying neurodegenerative processes. While dystonia is often observed as a symptom within these broader disease contexts, it is rarely the sole clinical manifestation. This suggests that autophagy and lysosomal dysfunction may contribute to dystonia as part of a more complex neurodegenerative phenotype, rather than serving as isolated causative mechanisms.

The homotypic fusion and protein sorting (HOPS) complex plays a critical role in the fusion of autophagosomes with lysosomes, a key step in the autophagy–lysosomal degradation pathway. Initially studied in the context of various neurodegenerative and lysosomal storage disorders, this pathway has more recently been implicated in dystonia, particularly through mutations in genes such as VPS11, VPS16, and VPS41 [42]. While these findings suggest a potential mechanistic link, they should be interpreted with caution. The HOPS complex represents a downstream component of the autophagy pathway, and its dysfunction may reflect broader neurodegenerative processes rather than dystonia-specific mechanisms. Moreover, many individuals in these studies presented with heterogeneous clinical phenotypes beyond dystonia, which may confound the interpretation of statistically significant associations and limit their clinical applicability.

It has long been hypothesized—but only recently investigated—that dystonia, despite affecting specific body regions or presenting with similar severity and age of onset, is unlikely to be caused by a single gene. Recent genome-wide association studies (GWASs) have confirmed this complexity, revealing no single-nucleotide polymorphisms (SNPs) consistently associated with isolated dystonia [43]. This finding underscores the substantial clinical and genetic heterogeneity of the disorder, which poses significant challenges for the design and interpretation of genetic studies. For example, the same clinical phenotype—such as dystonia-myoclonus—can result from multiple distinct genetic mutations. Conversely, individuals or families carrying the same genetic variant may exhibit variable penetrance and a wide range of phenotypic presentations, further complicating diagnosis, prognosis, and treatment strategies.

## 3. Insights from Animal Models

Neural space and developmental timing are closely interconnected. Disruptions occurring during critical periods of nervous system development can lead to long-lasting neurological disorders [44]. During neurodevelopment, the spinal cord, medulla, and pons are the first regions to mature, progressing through five key stages: neurogenesis, migration (including differentiation and neurite growth), gliogenesis, synaptogenesis, and myelination [45].

One limitation of traditional developmental theories is that they often overlook the unique maturation timelines of specific neural circuits, particularly those involved in motor control. These circuits develop and integrate at different rates depending on both spatial and temporal factors. As a result, variations in the timing and location of developmental disturbances can lead to diverse clinical presentations of dystonia, such as differences in body distribution (focal, segmental, generalized, hemidystonia) and age of onset (early vs. late) [46].

### 3.1. Critical Windows of Dystonia Pathogenesis

Reproducing critical developmental windows in experimental models requires precise control over three key factors: spatial specificity, temporal regulation, and methodological reversibility. Spatial specificity involves targeting distinct neural substrates, guided by molecular and genetic markers. Once these regions are identified, temporal regulation becomes essential to differentiate between early- and late-onset dystonia. This level of control allows researchers to investigate the genetic and epigenetic mechanisms that influence disease onset and progression. Importantly, reversibility is also crucial—enabling gene function to be restored within a defined window to assess the extent and permanence of developmental lesions.

Li et al. addressed this challenge by developing a mouse model to identify the critical window of pathogenesis in DYT1 dystonia, using the forebrain as the targeted region and employing a Tet-On system to regulate gene expression (Table 2) [47]. The forebrain was selected based on observations that mice with lesions in this area exhibited abnormal posturing when suspended by the tail. The Tet-On system functions by using the Tet repressor protein (TetR), which binds to the Tet operator (tetO) sequence to block transcription. Upon administration of tetracycline or its derivative doxycycline, this interaction is disrupted, allowing gene expression to resume.

Despite their utility, mouse models require significant time, financial resources, and specialized equipment. To address these limitations, Drosophila models have been developed as a more efficient alternative. While establishing a mouse model typically takes around three months, Drosophila models can be generated in just ten days. Dystonia-like motor symptoms in Drosophila are assessed using software that tracks the movement of their six limbs. Lowe et al. utilized this approach to investigate the critical period for motor dysfunction by overexpressing a gene associated with BK potassium channels, which are linked to dyskinesia [48]. They observed mild gait abnormalities when the gene was overexpressed during the mid-pupal stage and more severe symptoms during the late-pupal stage. Notably, no abnormalities were detected when overexpression occurred during embryonic, early pupal, or adult stages.

### 3.2. Neural Substrate of Dystonia

In the 1930s, Graham Brown’s laboratory in Cardiff developed a treadmill system to study locomotion and spinal networks. In a landmark experiment, they filmed a normal cat running on the treadmill, followed by a high-decerebrate cat—one with its cerebral hemispheres removed—demonstrating that basic locomotor patterns could be generated by spinal circuits alone [49]. These automatic limb movements, which resemble dystonic features, suggest that the spinal cord may play a central role in the pathophysiology of dystonia.

Building on this concept, Pocratsky et al. developed a dystonia model that specifically targeted the spinal cord while sparing the brain. Using the TOR1A gene and a FLP-FRT system (instead of the more common Cre-Lox), they achieved temporospatial control of gene knockdown via CDX2:FlpO, which is selectively expressed in the spinal cord and dorsal root ganglia. To further investigate the role of sensory input, they employed Advillin-Cre, which is expressed exclusively in sensory neurons. The resulting phenotype included hindlimb hyperextension within 48 h, followed by trunk instability by postnatal day 6, forelimb deficits by day 8, and involvement of the neck by day 10 [50]. This progression mirrors the clinical presentation of severe DYT1 (TOR1A) dystonia in humans [51]. Interestingly, when TOR1A was knocked down only in the sensory system using Advillin-Cre, the mice initially showed mild symptoms but later developed normally, suggesting a modulatory rather than primary role of sensory input. Similar findings were observed in a DYT12 (ATP1A3) model, where spinal cord dysfunction was associated with reduced activity and hyperpolarization of motor neurons [52].

Several physiological features commonly observed in dystonia models warrant further investigation. These include spontaneous muscle activity at rest and co-contraction during voluntary movement, which can be measured via electromyography (EMG) in the gastrocnemius muscle—specifically, 15 mm distal to the sciatic nerve trifurcation [53]. These measurements are critical, as dystonia is thought to involve impaired spinal inhibition [54], a phenomenon confirmed in the Pocratsky et al. study [50]. Additionally, delayed H-reflex recovery—particularly in patients with generalized dystonia—further supports the hypothesis of reduced spinal inhibitory control [55].

### 3.3. Lesioning of Brain Regions in Dystonia

Dystonia can be experimentally induced in wild-type animals through targeted brain lesioning techniques. For instance, injecting the mycotoxin 3-nitropropionic acid into the striatum of mice reduces GABAergic transmission, resulting in dystonia-like movements. In primates, peripheral administration of 1-methyl-4-phenyl-1,2,3,6-tetrahydropyridine (MPTP) leads to dopaminergic neuronal loss and a dystonia-like phenotype [56].

The cerebellum has also emerged as a key region in dystonia modeling. Excitatory stimulation using kainate can induce abnormal Purkinje cell firing, while lesioning the pedunculopontine nucleus—likely a relay between the cerebellum and basal ganglia—can also produce dystonia-like symptoms. Notably, mice with CACNA1A mutations exhibit worsened dystonic posturing following cerebellar lesions [57], and several studies have shown that cerebellar damage exacerbates dystonia in genetic models [58]. Conversely, surgical removal of cerebellar regions has been shown to alleviate dystonic attacks in rats with mutations in ATCAY, a gene encoding caytaxin, which is involved in cerebellar cortex development [59].

Further evidence of cerebellar involvement comes from a pharmacological mouse model of DYT/PARK-ATP1A3 dystonia. In this model, ouabain—a blocker of the α3-subunit of the sodium-potassium pump—was administered to cerebellar tissue, inducing generalized dystonia-like movements in wild-type mice [60]. To explore the underlying circuitry, bilateral lesions were applied to the centrolateral thalamus, which connects the cerebellum to the basal ganglia. These lesions significantly reduced dystonia-like symptoms. Similar improvements were observed with neurotoxic silencing of the centrolateral thalamus in the same model [61]. Interestingly, bilateral silencing of the motor cortex using tetrodotoxin produced only modest symptom relief, suggesting that cerebellar circuits may play a more central role in DYT/PARK-ATP1A3 dystonia than cortical pathways—possibly due to gene-specific modulation of cerebellar function.

A major limitation of basal ganglia lesioning models is their lack of cellular specificity, which reduces their translational relevance. Additionally, the role of peripheral structures—such as the spinal cord, cerebellum, and sensory systems—is often underappreciated. It is essential to further investigate whether these peripheral components contribute to the etiology of dystonia or merely modulate its progression.

### 3.4. Pathophysiology Basis of Dystonia

The convergence model of dystonia pathophysiology posits that multiple distinct pathways ultimately lead to a shared final common circuit responsible for symptom generation. In contrast, the divergence model suggests that a single neural circuit can give rise to a spectrum of movement disorders depending on how it is perturbed. Supporting this, a recent study demonstrated that stimulation of a single cerebellar nucleus—the interposed nucleus, which connects to the thalamus and midbrain—could produce ataxia, dystonia, or tremor depending on the nature of the disruption [62]. Specifically, silencing excitatory input from the inferior olive modeled dystonia, harmaline application to the cerebellar surface induced tremor, and inhibition of Purkinje cell output led to ataxia. The researchers identified three electrophysiological features that distinguished these phenotypes: skewness, coefficient of variation (CV2, reflecting spike-to-spike irregularity), and instantaneous firing rate (ISI). Dystonia was associated with high skewness and elevated ISI. Further modeling using parsed spike train patterns in Purkinje cells confirmed that dystonia was characterized by slow, irregular firing; tremor by fast, rhythmic discharges; and ataxia by continuous 50 Hz pulses.

Another critical dimension in dystonia pathophysiology is the role of the peripheral nervous system. In a DYT-TOR1A mouse model, peripheral nerve injury (e.g., sciatic nerve crush) accelerated the onset of dystonia, regardless of the body region affected. Genetically predisposed mice developed dystonia more frequently than wild-type controls following injury. Interestingly, the neural circuits involved in this injury-induced dystonia differed by genotype: in wild-type mice, the cerebellum was implicated, whereas in mutant mice, the cortex and striatum were more prominently involved [63]. Additional studies have revealed altered glucose metabolism in dystonic animals, with energy redistribution in motor and somatosensory cortices. Notably, treatment with fenofibrate—a peroxisome proliferator-activated receptor alpha (PPARα) agonist—failed to restore normal gene expression, suggesting persistent metabolic dysregulation [64].

Preterm birth is frequently associated with primary generalized dystonia, likely due to disruptions in neurodevelopmental processes. Animal models of preterm birth more commonly exhibit dystonic phenotypes and show impaired cortical inhibition, attributed to a reduction in parvalbumin-positive interneurons [65]. These findings underscore the importance of using developmental models to study the genetic and molecular mechanisms underlying dystonia, particularly those involving neurodevelopmental genes.

### 3.5. Limitations of Animal Models

While animal models have been instrumental in advancing our understanding of dystonia pathophysiology, they vary considerably in their ability to replicate the human phenotype. Most models fail to reproduce hallmark clinical features such as task-specificity, patterned co-contractions, and sensory tricks. Standard behavioral assays often lack the sensitivity and specificity needed to capture these complex motor patterns. Although rodent models—particularly genetically engineered mice with mutations in TOR1A or ATP1A3—offer high genetic fidelity and allow for temporospatial control of gene expression, their limited behavioral repertoire restricts translational relevance.

Drosophila models, despite their anatomical simplicity, offer rapid generation times and cost-effective platforms for high-throughput genetic screening. Lesion-based models in rodents and primates can induce dystonia-like movements but often lack disease specificity and rely on non-physiological mechanisms. Non-human primate models more closely mimic human motor behavior but are limited by ethical concerns, high costs, and reduced feasibility for genetic manipulation. Thus, no single model fully recapitulates the human dystonia phenotype. Each provides unique insights, underscoring the need for integrative approaches that combine genetic, developmental, and neurophysiological perspectives to enhance translational relevance.

Common behavioral assays used in animal models include tail suspension (to assess posturing), open field tests (for general activity), beam and pole climbing (coordination vs. strength), and rotarod or treadmill tests (coordination vs. speed). While these methods quantify gross motor performance, they often fail to capture the nuanced impairments seen in dystonia, such as altered limb kinematics or abnormal muscle contractions. Emerging technologies—such as high-throughput pose estimation using computer vision—offer promising solutions. These tools enable detailed analysis of interlimb and intralimb coordination, digit positioning, and grip strength, particularly in models of task-specific dystonia [66].

Electromyography (EMG) in mice has traditionally been challenging due to their small muscle size. However, the development of high-density EMG systems, such as Myomatrix Arrays, now allows for precise quantification of motor unit activity. These arrays support both quiet stance and functional recruitment assessments and enable longitudinal recordings for up to 60 days post-implantation, significantly enhancing the quality and reproducibility of electrophysiological studies [67].

## 4. Insights from Imaging Studies

### 4.1. Functional Connectivity Is Altered at the Regional Brain Level

Dystonia is increasingly recognized as a neural network disorder, with neuroimaging studies revealing distinct patterns of abnormal connectivity that may serve as biomarkers for diagnosis and treatment development. Early functional imaging studies demonstrated that network dysfunction in dystonia is not limited to dystonia-inducing tasks. Abnormal activation patterns were observed during tasks that did not provoke symptoms, as well as during somatosensory stimulation [68]. These studies revealed both hyperactivation and hypoactivation in various brain regions, though the directionality and clinical relevance of these changes were not always clear. Consistently, abnormalities have been reported in the basal ganglia, cerebellum, thalamus, and sensorimotor cortex—regions implicated across multiple dystonia subtypes, including cervical and laryngeal dystonia [69]. Notably, cortical alterations are more frequently observed in task-specific dystonias (e.g., hand and laryngeal dystonia), whereas subcortical changes are more prominent in non-task-specific forms (e.g., cervical dystonia and blepharospasm) [70].

Advancements in neuroimaging techniques have enabled more detailed investigations into dystonia-related brain networks. Functional connectivity studies have shown that even during asymptomatic tasks, regional connectivity is altered. For example, in writer’s cramp, increased connectivity was observed between left and right Brodmann area 6 during writing, and between left Brodmann area 6 and the anterior supplementary motor area during tapping. During muscle contraction, four key regions were involved: bilateral Brodmann area 6, the anterior supplementary motor area, and the putamen [71]. Similar findings have been reported in laryngeal dystonia, where enhanced connectivity was observed between the globus pallidus and ventral thalamus during syllable production, and among the putamen, globus pallidus, and ventral thalamus during whimpering [72].

Importantly, functional abnormalities have also been detected at rest, without any task engagement. In cervical dystonia, disruptions in the sensorimotor and frontoparietal networks have been documented [73]. Writer’s cramp has been associated with altered connectivity in the sensorimotor and default mode networks [74], while laryngeal dystonia shows dysfunction in sensorimotor and frontoparietal circuits [75]. These findings suggest that regional, rather than global, functional connectivity—particularly involving the striatal and primary sensorimotor cortex networks—may underlie isolated focal dystonias, including cranial, cervical, laryngeal, and limb subtypes [76].

### 4.2. Functional Connectivity Is Altered at the Whole-Brain Level

Growing knowledge of brain network architecture has led to expanded investigations into dystonia at the whole-brain level. Both regional and global network interactions have been analyzed in terms of their integration, efficiency, and organization [77]. These networks are often characterized using metrics such as clustering coefficients and hub connectivity, which help identify how information is processed and distributed across large-scale brain systems [78]. Brain regions with shared functional roles tend to form clusters, or “communities,” that support specific behaviors. The concept of network segregation—how these communities form and interact—has proven essential in understanding motor control. Notably, age influences whole-brain connectivity patterns, with distinct differences observed between younger (20–34 years) and older (65–89 years) adults. Another key factor is nodal influence, which refers to the strength and degree of connectivity in specific brain regions. Highly connected nodes, or “hubs,” are central to neural communication and integration [79].

Resting-state functional connectome studies in focal dystonia have revealed significant alterations in these network communities. In healthy individuals, the brain is typically organized into five major communities spanning the prefrontal, occipital, thalamic, cerebellar, and basal ganglia regions. In contrast, individuals with task-specific dystonia (e.g., writer’s cramp, laryngeal dystonia) and non-task-specific dystonia (e.g., blepharospasm, cervical dystonia) exhibit disorganized cortical communities and disrupted basal ganglia–thalamo–cerebellar networks [80].

During symptomatic task performance, these disruptions become more pronounced. In laryngeal dystonia, for example, neural communities are more spatially dispersed, and novel communities emerge that are not seen in healthy controls [81]. Additionally, key network hubs—regions critical for information transfer—are often lost or replaced. This reorganization particularly affects the primary sensorimotor cortex, parietal cortex, and thalamus. In some cases, these disrupted hubs are replaced by “provincial hubs,” which are less central and form a less efficient network architecture [82]. Similar patterns have been observed in writer’s cramp during asymptomatic tasks, such as sequence tapping with the unaffected hand, where hub loss and gain were noted in the primary motor cortex, thalamus, and cerebellum [83].

Network alterations also vary by dystonia phenotype and genotype. For instance, abductor versus adductor laryngeal dystonia shows distinct changes in parietal hub connectivity [82]. In writer’s cramp, differences in cerebellar connectivity have been observed between simple and complex phenotypes [83].

The concept of a “network kernel” has been introduced to describe brain regions most consistently implicated in dystonia, including the cerebellum, basal ganglia, and thalamus. In task-specific dystonias (e.g., writer’s cramp, musician’s focal hand dystonia, laryngeal dystonia), decreased activity is observed in the primary motor, parietal, medial frontal, occipital cortices, and cerebellum. Conversely, increased activity is seen in the primary somatosensory cortex, parietal cortex, occipital cortex, thalamus, and cerebellum. Interestingly, both dystonia patients and healthy controls share common hubs, such as the primary somatosensory cortex, parietal operculum, medial frontal gyrus, and occipital cortex. However, the network kernel is modulated by the affected body region and behavioral context. For example, focal hand dystonia shows altered connectivity in motor control networks, while laryngeal dystonia involves sensorimotor processing circuits. Similarly, musician’s dystonia is characterized by disrupted sensory–motor execution circuits, whereas non-musician dystonia involves abnormal integration of sensory feedback into motor planning [84].

Although genotypes such as DYT1 and DYT6 are believed to influence the network kernel, current evidence remains limited [85]. It is also important to note that most neuroimaging studies focus on focal dystonias—particularly task-specific and laryngeal forms—due to their reproducibility in fMRI protocols. As a result, these findings may not generalize to generalized dystonia, which may involve distinct mechanisms. This limitation is further supported by PET studies showing variable metabolic patterns across brain regions in different dystonia types [86], reinforcing the heterogeneity of dystonia-related network alterations.

### 4.3. Dystonic Neural Network and Pathophysiological Mechanisms

Functional connectivity analyses have significantly advanced our understanding of dystonia by revealing temporal correlations between spatially distinct brain regions. However, while functional connectivity describes statistical associations, it does not clarify the direction or causality of these interactions. To address this, studies have begun to explore effective connectivity, which evaluates the influence that one neural element exerts over another. This approach is essential for distinguishing between primary pathological mechanisms and compensatory network adaptations. By differentiating between hemodynamic and neuronal signals, effective connectivity can help determine whether observed abnormalities are causative or reactive in nature.

Effective connectivity in dystonia has been investigated using dynamic causal modeling (DCM) during resting-state fMRI. One study examined a fully connected model involving the right and left premotor cortices, the left inferior parietal cortex, and the left putamen. In patients with laryngeal dystonia, compared to healthy controls, there was abnormal bidirectional connectivity between the left inferior parietal cortex and the right premotor cortex. The analysis revealed top-down hyperexcitability from the left inferior parietal cortex to the left putamen, as well as interhemispheric hyperexcitability from the right to the left premotor cortex—suggesting a dysregulated sensorimotor integration network [87]. Complementary EEG studies have also shown abnormal gamma-band activity and hyperconnectivity between the superior parietal and middle frontal cortices [88].

In writer’s cramp, effective connectivity was similarly assessed using DCM during asymptomatic sequence tapping with the unaffected hand. The results showed deficient connectivity between the pallidum and primary motor cortex, as well as between the primary motor cortex and putamen. In contrast, increased connectivity was observed between the cerebellum and both the motor cortex and basal ganglia, indicating a possible compensatory role of cerebellar circuits [89]. Another EEG study further identified abnormal connectivity between the premotor and right primary motor areas, characterized by dysregulated beta and gamma band activity. Specifically, there was increased excitatory bidirectional coupling between the left premotor and primary motor cortices at low frequencies, along with heightened inhibitory influence from the supplementary motor area to the premotor cortex in the beta and gamma bands [90].

### 4.4. Imaging in Genetics, Biological Factors, and Environmental Triggers

There is growing evidence that dystonia involves a polygenic risk architecture, with implicated genes associated with synaptic function, neuronal projections, synaptic transmission, and neurodevelopment. In laryngeal dystonia, these genes are particularly linked to the premotor and primary sensorimotor cortices, as well as the inferior parietal cortex [91]. Additionally, neuroimaging studies in patients with DYT1, DYT6, and non-manifesting carriers have revealed abnormal glucose metabolism in regions such as the parietal cortex, precuneus, thalamus, cerebellum, and brainstem [92]. Interestingly, in laryngeal dystonia, individuals with genetic penetrance but no clinical symptoms show different patterns of regional involvement compared to symptomatic patients. However, both groups share functional alterations in the left caudate nucleus, suggesting its potential role in the disorder’s pathophysiology [93].

Epidemiological data also indicate that dystonia prevalence varies by sex, race, and environmental exposures. For example, laryngeal dystonia is more common in females, individuals of white race, professional voice users, and those with a history of recurrent upper airway infections [94]. Neuroimaging studies have identified neural correlates of these extrinsic risk factors, including alterations in the premotor, parietal, insular, and striatal regions [95].

Although neuroimaging research in dystonia has advanced considerably, it remains an evolving field. Early insights came from PET studies in laryngeal dystonia, which revealed metabolic and functional abnormalities [96]. Since then, the development of advanced imaging modalities—such as functional MRI (fMRI), diffusion tensor imaging (DTI), and voxel-based morphometry—has enabled more precise mapping of structural and functional changes across dystonia subtypes. Despite these advances, several challenges persist, including the identification of consistent biomarkers, differentiation between primary and secondary dystonia, and the integration of motor and non-motor network alterations. Future research that combines multimodal imaging with genetic and electrophysiological data holds promise for a more comprehensive understanding of dystonia pathophysiology and the development of more targeted diagnostic and therapeutic strategies.

### 4.5. Beyond Abnormal Neural Networks

The identification of abnormal neural networks in patients with dystonia has been greatly facilitated by advances in neuroimaging techniques. Notably, ultra-high-field MRI studies have revealed atypical somatosensory representations of the fingers in individuals with dystonia. These studies demonstrated a disproportionate increase in activity within the input layers (cortico-cortical layers II/III) relative to the output layers (cortico-spinal layers Vb/VI) of the cortex [97]. Such spatial resolution was previously achievable only in animal models.

Morphometric analyses have also contributed valuable insights into the pathophysiology of dystonia, particularly in relation to neural plasticity associated with overuse or underuse. For example, patients with writer’s cramp often exhibit increased gray matter volume in the ipsilateral hemisphere, potentially reflecting compensatory cerebellar mechanisms [98]. However, contrasting findings have also been reported, with some studies indicating a reduction in gray matter volume [99], underscoring the heterogeneity of structural brain changes in dystonia.

## 5. Potential Treatment Targets

Current therapeutic strategies for dystonia primarily aim to modulate disrupted synaptic transmission, particularly within the GABAergic, dopaminergic, and cholinergic systems. The choice of pharmacological intervention often depends on the specific dystonia subtype. However, emerging evidence suggests that future drug development should also target cellular stress responses and lysosomal pathways. Compounds under investigation for Parkinson’s disease, such as trehalose (an autophagy activator) [100] and eliglustat (a substrate reduction therapy) [101], may hold therapeutic potential for dystonia as well. Additionally, modulators of the integrated stress response—including inhibitors like 2BAct and potentiators such as ritonavir, Sephin1, and Raphin1—represent promising candidates for further exploration [102].

Mitochondrial dysfunction, observed in certain dystonia subtypes, presents another potential therapeutic target. Agents that restore redox homeostasis warrant investigation in these cases. Furthermore, although less frequently discussed, the role of heavy metal accumulation—particularly iron—remains unclear. While iron buildup is associated with neurodegeneration, it is not yet established whether it contributes to the pathogenesis of dystonia or merely reflects downstream neurodegenerative processes.

Spinal cord stimulation (SCS) is another area of interest, particularly in dystonia models with spinal cord-related pathophysiology. Although earlier studies on SCS yielded inconsistent results, these investigations often relied on outdated techniques and materials [103]. In contrast, deep brain stimulation (DBS)—which targets central brain circuits—has demonstrated the capacity to induce neuroadaptive changes across multiple networks, potentially including secondary effects on spinal pathways. Historically, ablative procedures such as thalamotomy and pallidotomy were effective in treating generalized dystonia and have since been largely replaced by DBS. The internal segment of the globus pallidus (GPi) remains the primary DBS target, with particularly robust responses observed in primary generalized dystonia. However, other dystonia subtypes often show less favorable outcomes, suggesting variability in the underlying neural circuitry [104]. Given these findings, epidural and transcutaneous spinal stimulation may offer novel avenues for modulating neuroplasticity in dystonia and merit further investigation.

A major challenge in dystonia research is the heterogeneity of experimental protocols, including the use of diverse animal models and the targeting of different neuroanatomical structures. Standardization of drug targets and consistent definitions of dystonia are essential to improve the comparability and reproducibility of findings.

Finally, therapeutic interventions monitored via neuroimaging are typically assessed at the whole-brain level. This approach may obscure localized network changes relevant to treatment. A proposed solution involves the segregation of functional networks to reduce interpretive bias. In this context, the development of ultra-high-field MRI holds significant promise for refining our understanding of dystonia-related networks and guiding future therapeutic strategies.

## 6. Future Studies

Recent efforts by the global scientific community have led to new classification systems for Parkinson’s disease, incorporating neuropathological findings, patterns of neurodegeneration, and pathogenic gene variants [105]. In contrast, dystonia classification remains largely phenomenological, based on clinical presentation and body distribution. Given the substantial genetic discoveries over the past decade, there is a compelling need to develop a new classification framework for dystonia. Such a system could integrate factors including neurodevelopmental versus neurodegenerative origins, pathogenic gene variants, and involvement of molecular pathways such as the integrated stress response and lysosomal function.

A critical area for future research is the timing of point mutations associated with dystonia, particularly in cases involving genes implicated in abnormal neurodevelopment. Identifying the optimal developmental window during which these mutations exert their effects is essential. For example, in Niemann–Pick type C (NPC) dysfunction, dystonia appears within a specific temporal window. Moreover, certain injuries may activate these genes, though the mechanisms and variable penetrance remain poorly understood.

The discovery of novel dystonia-related genes through advanced genomic techniques raises important questions about their interconnectivity. It is unlikely that each individual harbors a unique mutation leading to a similar clinical phenotype. Therefore, future studies should investigate shared molecular pathways among these genes. Computational tools and network-based models may be instrumental in elucidating these relationships.

Despite progress in identifying molecular abnormalities underlying dystonia, significant gaps remain in our understanding of how these changes lead to abnormal motor control. Animal models are essential for bridging this gap, yet current models suffer from limitations in homogeneity, reproducibility, and spatiotemporal resolution. The development of more refined and representative models is critical for advancing the field.

The role of the spinal cord in dystonia pathogenesis also warrants further investigation. While some individuals exhibit basal ganglia abnormalities, it is unclear whether these are primary or secondary to disrupted spinal–brain connectivity. In mouse models of dystonia, restoration of TOR1A function led to only partial recovery, suggesting that brain regions contribute to the modulation of dystonic symptoms. Future models should target specific brain regions rather than relying solely on generalized or spinal cord-focused approaches.

In cases of dystonia refractory to deep brain stimulation (DBS), the cerebellar pathways—particularly those involving the interposed nuclei—may represent alternative therapeutic targets. This approach may be most appropriate for individuals without a known genetic etiology but with identifiable electrophysiological abnormalities. In dystonic mouse models with disrupted sleep architecture, cerebellar-targeted DBS improved both motor symptoms and sleep quality [106].

Advancements in neuroimaging are essential for further elucidating dystonia-related network dysfunction. Most current studies provide broad observations of pathophysiology without identifying specific mechanisms or therapeutic targets. Future imaging research should be closely aligned with treatment strategies, including pharmacological and neuromodulatory interventions. Artificial intelligence (AI)-driven diagnostic tools, such as those leveraging raw structural MRI data and platforms like DystoniaNet, offer promising avenues for objective diagnosis and personalized treatment planning. These tools may also facilitate outcome prediction for therapies such as botulinum toxin injections and DBS, enabling more precise targeting based on individual network profiles. Ultimately, this could lead to the development of closed-loop adaptive systems that deliver individualized, real-time treatment for dystonia.

While genetic discoveries have significantly advanced our understanding of dystonia, growing evidence highlights the importance of environmental and epigenetic factors in modulating phenotypic expression. Traumatic brain injury, perinatal hypoxia, infections, and exposure to dopamine antagonists have all been implicated as potential triggers in genetically predisposed individuals. These environmental insults may influence gene expression through epigenetic mechanisms such as DNA methylation, histone modification, and non-coding RNA regulation, thereby altering neural circuitry and motor function. For instance, individuals with TOR1A or THAP1 mutations may remain asymptomatic until exposed to specific environmental stressors. Future research integrating genomic, epigenomic, and exosomic data will be essential to unravel the complex interplay between inherited susceptibility and environmental influences in dystonia pathogenesis.

## 7. Conclusions

Advancements in genomic technologies have significantly expanded the number of genes associated with dystonia. The transition from exome to whole-genome sequencing is expected to further accelerate the discovery of novel genetic contributors. Notably, there is considerable overlap between genes implicated in neurodevelopment and those associated with dystonia, underscoring the importance of investigating these genes in greater depth—particularly in relation to their pathophysiological mechanisms and potential as therapeutic targets.

Dystonia is increasingly recognized as a disorder of neural networks, with alterations detectable at both regional and whole-brain levels. The interplay between genotype and phenotype plays a critical role in shaping the architecture and dynamics of the dystonic network, which underlies the clinical heterogeneity observed across patients. Within this framework, the concepts of a network core and a more refined network kernel have been proposed to describe the long-range and focal disruptions in connectivity, respectively. These network alterations, along with their interactions, contribute to the emergence of complex and specific motor behaviors.

Furthermore, dystonia-specific neuroimaging holds promise for the development of objective diagnostic tools and personalized treatment strategies. As our understanding of the molecular and network-level underpinnings of dystonia continues to evolve, these insights will be instrumental in guiding the next generation of therapeutic interventions.

## Figures and Tables

**Table 1:** Genetic Profile Of Dystonia in Population Studies.

Country	NGS Type ^a^	Genetic Dystonia ^b^	n ^c^	Percentage ^d^	Comment	Reference
Australia	Genome	13	111	11.7	Most were isolated dystonia (focal, multifocal, segmental, generalized), but some had dystonia-parkinsonism and myoclonus-dystonia.	Kumar et al. (2019) [3]
France	Exome	11	32	34	Most were complex dystonia (dystonia with ataxia, deafness, epilepsy, and intellectual disability). Other patients had isolated dystonia and dystonia combined with other movement disorders (myoclonus, chorea, and tic).	Wirth et al. (2020) [4]
Multiple European countries ^e^	Exome	135	708	19	Isolated and combined dystonia, with or without associated symptoms, including cases clinically diagnosed as dystonic cerebral palsy.	Zech et al. (2020) [5]
South Korea	Exome	9	43	21	Isolated dystonia, dystonia co-occurring with other movement disorders, and dystonia associated with neurological or systemic manifestations.	Ahn et al. (2023) [6]
India	Exome	15	65	23	Generalized dystonia was the most common. Isolated dystonia and combined dystonia (chorea, parkinsonism, and myoclonus) were also investigated.	Dhar et al. (2024) [7]
Turkey	Exome	15	42	36	Isolated and combined dystonia (developmental delay, cognitive impairment, epilepsy, ataxia, choreoathetosis, rigidity, and bradykinesia).	Atasu et al. (2024) [8]
India	Exome	54	267	20%	Most were isolated dystonia. Others were combined and complex dystonia.	Saini et al. (2025) [9]
Multiple European countries ^e^ and Malaysia	Exome	153	1895	8	Most were isolated dystonia, but a small percentage of patients with additional neurological disorder were also included.	Thomsen et al. (2024) [10]
China	Exome	30	42	71	Cervical dystonia	Wu et al. (2024) [11]

^a^ Next-generation sequencing, including whole-genome or whole-exome, was performed. ^b^ Number of individuals that, after the next-generation sequencing was performed, received a formal diagnosis of a genetic dystonia. ^c^ Number of individuals with a phenomenological diagnosis of dystonia. ^d^ The percentage was obtained by quotient between the number of individuals with a genetic diagnosis and the number of individuals with a phenomenological diagnosis. ^e^ Austria, Czech Republic, France, Germany, Poland, Slovakia, and Switzerland.

**Table 2:** Critical window for the development of dystonia, based on Li et al. (2021) [47].

Period	Life Days	Definition	Finding
Embryonic	0	TOR1A suppressed during conception.	All mice developed abnormal posturing.
Adulthood	70	TOR1A suppressed during adulthood.	No mice developed abnormal posturing.
Early restore	21	TOR1A was suppressed during conception and restored function early.	There was a significant reduction in the time of abnormal posturing when compared to those that the function was not restored.
Late restore	70	TOR1A was suppressed during conception and restored function later after the phenotype became penetrant.	There was no significant benefit in restoring later the TOR1A function.
Acute versus chronic	21	TOR1A was restored early, and the restoration time was maintained between acute (from 21 to 70 days) and chronic (from 21 to 168 days).	There was no significant difference between maintenance of TOR1A restoration above 70 days.

## Data Availability

No new data created.

## Supplementary Materials

The following supporting information can be downloaded at https://www.mdpi.com/article/10.3390/brainsci15070767/s1, Table S1: Timeline of Gene Discovery in Dystonia [107,108,109,110]; Table S2: Phenotype–Gene Relationships of Dystonia in OMIM; Table S3: Overview of the genetic classification of dystonia according to MDSGene; Table S4: Genetic intersection between dystonia and neurodevelopmental disorders; Table S5: Converging Genes And Causative Pathways.
