# Supplementary material for: Neurobiology of Dystonia: Review of Genetics, Animal Models, and Neuroimaging
Source: Brain Sci. 2025 Jul 19;15(7):767. doi: 10.3390/brainsci15070767 (PMC12294012; doi:10.3390/brainsci15070767)
Supplement: Supplementary file 1 [file brainsci-15-00767-s001.zip › brainsci-3741237-supplementary.pdf]

Supplementary Material

Tables

Table S1. Timeline Of Gene Discovery In Dystonia.

Table S2. Phenotype-Gene Relationships Of Dystonia in OMIM.

Table S3. Overview of the genetic classification of dystonia according to MDSGene.

Table S4. Genetic intersection between dystonia and neurodevelopmental disorders.

Table S5. Converging Genes And Causative Pathways.

Table S1

Table S1. Timeline Of Gene Discovery In Dystonia.	
Year	Discovery
1993	Linkage mapping of dopa-responsive dystonia (DRD) to chromosome 14q. Nygaard et al. (1993) [107]
1994	GCH1 (DYT5a). Childhood-onset dystonia, diurnal fluctuation, reduced penetrance, dramatic response to levodopa. RA et al. (1993) [108]
1995	TH (DYT5b)
1997	TOR1A (DYT1). Early-onset dystonia, generalized dystonia, reduced penetrance, and reasonable response to DBS. Ozelius et al. (1997) [109]
2001	SGCE (DYT11)
2004	MR-1 (DYT8)
2004	ATP1A3 (DYT12)
2007	TAF1 (DYT3)
2008	SLC2A1 (DYT18)
2009	THAP1 (DYT6). Early-onset dystonia, craniocervical predominance, reduced penetrance, and variable DBS response. Fuchs et al. (2009) [110]
2010	Next-generation sequencing (NGS) development
2011	PRRT2 (DYT10)
2012	CIZ1 (DYT23)
2012	ANO3 (DYT24). Early/late-onset dystonia, craniocervical dystonia, generalized dystonia.
2012	GNAL (DYT25). Early/late-onset dystonia, craniocervical dystonia, and generalized dystonia.
2013	TUBB4 (DYT4). Early-onset dystonia, generalized dystonia, dysphonia, and more complex phenotypes.
2016	KCTD17, HPCA, MECR, KMT2B
2020	VPS16
2022	TMEM151A, AOPEP, KCNN2, EIF2AK2, ATP5MC3, ATP5F1B
2024	SHQ1, NUP54, and many others

Table S2

Table S2. Phenotype-Gene Relationships Of Dystonia in OMIM[a]								
Nomenclature	Phenotype	Location	Phenotype number	Inheritance	Phenotype mapping key	Gene/ Locus	Gene/ Locus MIM number	Gene ID[b]
DYT1	Dystonia-1, torsion	9q34.11	128100	AD	3	Torsin family 1 member A (TOR1A)	605204	1861
DYT2	Dystonia 2, torsion, autosomal recessive	1p35.1	224500	AR	3	Hippocalcin (HPCA)	142622	3208
DYT3	Dystonia-Parkinsonism, X-linked	Xq13.1	314250	XLR	3	TATA-box binding protein associated factor 1 (TAF1)	313650	6872
DYT4	Dystonia 4, torsion, autosomal dominant	19p13.3	128101	AD	3	Tubulin beta 4A class IVa (TUBB4A)	602662	10382
DYT5	Dystonia, DOPA-responsive	14q22.2	128230	AD, AR	3	GTP cyclohydrolase 1 (GCH1)	600225	2643
DYT6	Dystonia 6, torsion	8p11.21	602629	AD	3	THAP Domain Containing 1 (THAP1)	609520	55145
DYT7	Dystonia-7, torsion	18p	602124	AD	2	NA	NA	NA
DYT8	Paroxysmal nonkinesigenic dyskinesia 1	2q35	118800	AD	3	PNKD metallo-beta-lactamase domain containing (PNKD)	609023	25953
DYT9	Dystonia 9	1p34.2	601042	AD	3	Solute carrier family 2 member 1 (SLC2A1)	138140	6513

DYT10	Episodic kinesigenic dyskinesia 1	16p11.2	128200	AD	3	Proline rich transmembrane protein 2 (PRRT2)	614386	112476
DYT11	Dystonia-11, myoclonic	7q21.3	159900	AD	3	Sarcoglycan epsilon (SGCE)	604149	8910
DYT12	Dystonia-12	19q13.2	128235	AD	3	ATPase Na+/K+ transporting subunit alpha 3 (ATP1A3)	182350	478
DYT13	Dystonia 13, torsion	1p36.32-p36.13	607671	AD	2	NA	NA	NA
DYT14	It was originally described based on GCH1 gene, but later it was grouped with DYT5							
DYT15	Dystonia-15, myoclonic	18p11	607488	AD	2	NA	NA	NA
DYT16	Dystonia 16	2q31.2	612067	AR	3	Protein activator of interferon induced protein kinase EIF2AK2 (PRKRA)	603424	8575
DYT17	Dystonia-17, primary torsion	20p11.2-q13.12	612406	AR	2	NA	NA	NA
DYT18	GLUT1 deficiency syndrome 2, childhood onset	1p34.2	612126	AD	3	Solute carrier family 2 member 1 (SLC2A1)	138140	6513
DYT19	Episodic kinesigenic dyskinesia 2	16q13-q22.1	611031	AD	2	NA	NA	NA
DYT20	Paroxysmal nonkinesigenic dyskinesia 2	2q31	611147	AD	2	NA	NA	NA
DYT21	Dystonia 21	2q14.3-q21.3	614588	AD	2	NA	NA	NA
DYT22	This record was withdrawn by the Human Genome Organisation (HUGO) Gene Nomenclature Committee (HGNC).							
DYT23	Dystonia 23	9q34	614860	AD	2	NA	NA	NA

DYT24	Dystonia 24	11p14.3-p14.2	615034	AD	3	Anoctamin 3 (ANO3)	610110	63982
DYT25	Dystonia 25	18p11.21	615073	AD	3	G protein subunit alpha L (GNAL)	139312	2774
DYT26	Dystonia 26, myoclonic	22q12.3	616398	AD	3	Potassium channel tetramerization domain containing 17 (KCTD17)	616386	79734
DYT27	Dystonia 27	2q37.3	616411	AR	3	Collagen type VI alpha 3 chain (COL6A3)	120250	1293
DYT28	Dystonia 28, childhood-onset	19q13.12	617284	AD	3	Lysine methyltransferase 2B (KMT2B)	606834	9757
DYT29	Dystonia, childhood-onset, with optic atrophy and basal ganglia abnormalities	1p35.3	617282	AR	3	Mitochondrial trans-2-enoyl-CoA reductase (MECR)	608205	51102
DYT30	Dystonia 30	20p13	619291	AD	3	Vacuolar protein sorting-associated protein 16 (VPS16)	608550	64601
DYT31	Dystonia 31	9q22.32	619565	AR	3	Aminopeptidase O (AOPEP)	619600	84909
DYT32	Dystonia-32	11q23.3	619637	AR	3	Vacuolar protein sorting-associated protein 11 (VPS11)	608549	55823
DYT33	Dystonia 33 (provisionally)	2p22.2	619687	AD, AR	3	Eukaryotic translation initiation factor 2 alpha kinase 2 (EIF2AK2)	176871	5610
DYT34	Dystonia 34, myoclonic (provisionally)	5q22.3	619724	AD	3	Potassium calcium-activated channel subfamily N	605879	3781

						member 2 (KCNN2)		
DYT35	Dystonia 35, childhood-onset (provisionally)	3p13	619921	AR	3	SHQ1	613663	55164
DYT36	Episodic kinesigenic dyskinesia 3	11q13.2	620245	AD	3	Transmembrane protein 151A (TMEM151A)	620108	256472
DYT37	Dystonia 37, early-onset, with striatal lesions	4q21.1	620427	AR	3	Nucleoporin 54 (NUP54)	607607	53371

Abbreviation: AD, autosomal dominant; AR, autosomal recessive; NA, not available/ not applicable; XLR, X-linked recessive.
[a] Information obtained from OMIM® database (https://www.omim.org/)
[b] Information obtained from NIH Gene database (https://www.ncbi.nlm.nih.gov/gene/)

Table S3

Table S3. Overview of the genetic classification of dystonia according to MDSGene[a]		
Monogenic forms	Isolated dystonia	DYT-ANO3; DYT-EIF2AK2; DYT-GNAL; DYT-HPCA; DYT-KMT2B; DYT-PRKRA; DYT-THAP1; DYT-TOR1A; DYT-VPS16; DYT-AOPEP
	Combined dystonia	DYT-COX20; DYT-DNAJC12; DYT-SLC39A14; DYT/PARK-ATP1A3; DYT/PARK-GCH1; DYT/PARK-TAF1; DYT/PARK-TH; DYT/CHOR-GNAO1; MYC/DYT-KCTD17; MYC/DYT-SGCE
	Complex dystonia	DYT-ACTB; DYT-ATP7B; DYT-BCAP31; DYT-DCAF17-(NBIA); DYT-DDC; DYT-FITM2; DYT-IRF2BPL; DYT-MECR; DYT-mt-ND6; DYT-OPA1; DYT-PANK2-(NBIA); DYT-SERAC1; DYT-SLC19A3; DYT-SUCLA2; DYT-TIMM8A; DYT-TUBB4A; DYT-VAC14; DYT/CHOR-ACAT1; DYT/CHOR-ADAR1; DYT/CHOR-FOXG1; DYT/CHOR-GCDH; DYT/CHOR-HPRT; DYT/CHOR-MUT; DYT/CHOR-PCCA/PCCB; DYT/PARK-CP-(NBIA); DYT/PARK-GLB1; DYT/PARK-PLA2G6-(NBIA); DYT/PARK-PTS; DYT/PARK-QDPR; DYT/PARK-SLC6A3; DYT/PARK-SLC30A10; DYT/PARK-SPR; ATX/DYT-SQSTM1
Disorders that usually present with other phenotypes but can have predominant dystonia		ATX-ATXN3; HSP-C19orf12-(NBIA); HSP/ATX-FA2H-(NBIA); HSP/ATX-KIF1C; CHOR-FTL-(NBIA); PARK-DNAJC6; PARK-WDR45-(NBIA)
List of genes causing neurodevelopmental delay and dystonia[b]		ARX; CTNNB1; GNB1; SUCLG1; VPS41; YY1

Abbreviations: ATX, ataxia; CHOR, chorea; DYT, dystonia; HSP, hereditary spastic paraplegia; NBIA, neurodegeneration with brain iron accumulation; PARK, Parkinson's disease.

[a] Information obtained from https://www.mdsgene.org/

[b] Conditions where less prominent dystonia can be encountered in the setting of predominant developmental disorders or epileptic encephalopathy

Table S4

Genetic intersection between dystonia and neurodevelopmental disorders
The data regarding the genes related to dystonia and neurodevelopmental disorders was obtained from the OMIM® database (https://www.omim.org/). The searched terms were “dystonia and neurodevelopmental disorder.”

Table S4 - Genetic intersection between dystonia and neurodevelopmental disorders		
Search	Term	Entries
dystonia	Search: '"dystonia" (Search in: Entries with: Genemap; Retrieve: gene map)'	472
neurodevelopmental disorder	Search: '"neurodevelopmental disorder" (Search in: Entries with: Genemap; Retrieve: gene map)'	586

The ratio of dystonia found in neurodevelopmental disorder genes was 76.62% (472/586).

Table S5

Converging Genes And Causative Pathways

STRING (Search Tool for the Retrieval of Interacting Genes/Proteins) is a comprehensive database exploring protein-protein interactions (PPIs). It integrates data from multiple sources, including experimental studies, computational predictions, and text mining, to build interaction networks that help researchers understand protein functions and biological pathways. STRING provides insights into direct (physical) and indirect (functional) associations between proteins, aiding in systems biology, disease research, and drug discovery. The platform also includes tools for functional enrichment analysis, allowing users to identify key pathways and molecular mechanisms relevant to their proteins of interest.

https://string-db.org/

We selected multiple proteins, and in the list of proteins, we included “EIF2AK2, EIF4A2, NUP54, PRKRA, THAP1, and TOR1A.” The network statistics showed six nodes, three edges, one average node degree, 0.33 average local clustering coefficient, zero expected number of edges, and 0.00141 protein-protein interaction enrichment p-value. The figures and table described below were rendered from STRING v.12

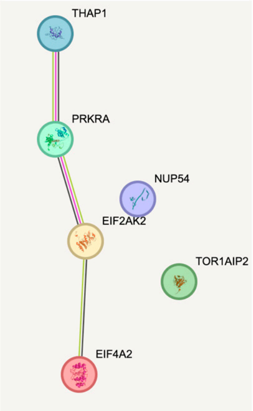

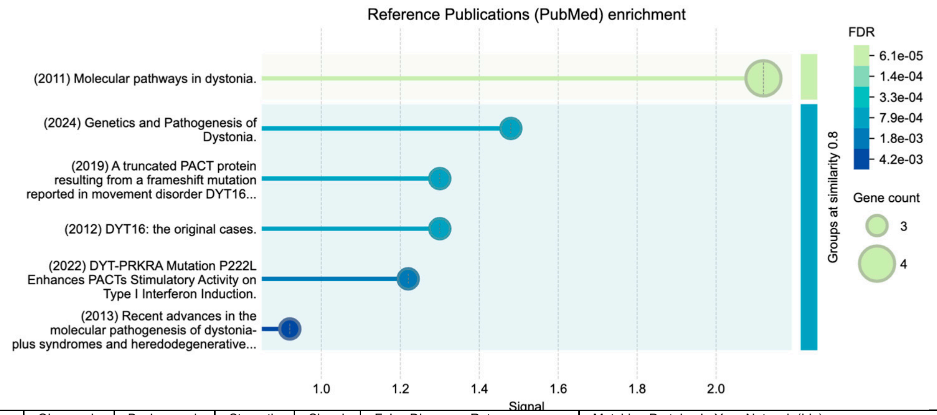

#Term ID	Term Description	Observed Gene Count	Background Gene Count	Strength	Signal	False Discovery Rate	Matching Proteins In Your Network (Ids)	matching proteins in your network (labels)
PMID:21134457	(2011) Molecular pathways in dystonia.	4	31	2.63	2.12	0.00062	9606.ENSP00000233057,9606.ENSP00000254250,9606.ENSP00000318176,9606.ENSP00000356584	EIF2AK2,THAP1,PRKRA,TOR1AIP2
PMID:37738511	(2024) Genetics and Pathogenesis of Dystonia.	3	8	3.09	1.48	0.0065	9606.ENSP00000233057,9606.ENSP00000254250,9606.ENSP00000318176	EIF2AK2,THAP1,PRKRA
PMID:31246344	(2019) A truncated PACT protein resulting from a frameshift mutation reported in movement disorder DYT16 triggers caspase activation and apoptosis.	3	12	2.91	1.3	0.0119	9606.ENSP00000233057,9606.ENSP00000254250,9606.ENSP00000318176	EIF2AK2,THAP1,PRKRA
PMID:22842711	(2012) DYT16: the original cases.	3	12	2.91	1.3	0.0119	9606.ENSP00000233057,9606.ENSP00000254250,9606.ENSP00000318176	EIF2AK2,THAP1,PRKRA

PMID:35625640	(2022) DYT-PRKRA Mutation P222L Enhances PACTs Stimulatory Activity on Type I Interferon Induction.	3	16	2.79	1.22	0.0152	9606.ENSP00000233057,9606.ENSP00000254250,9606.ENSP00000318176	EIF2AK2,THAP1,PRKRA
PMID:23814535	(2013) Recent advances in the molecular pathogenesis of dystonia-plus syndromes and heredodegenerative dystonias.	3	25	2.6	0.92	0.0428	9606.ENSP00000233057,9606.ENSP00000254250,9606.ENSP00000318176	EIF2AK2,THAP1,PRKRA

A second search was performed with some of the genes related to autophagy and lysosomal pathways. We selected multiple proteins, and in the list of proteins, we included “HEXA, IRF2BPL, NPC1, PINK1, PRKN, SPG11, TECPR2, VPS16, and WDR45.” The network statistics showed nine nodes, eight edges, 1.78 average node degree, 0.46 average local clustering coefficient, zero expected number of edges, and 7.23e-09 protein-protein interaction enrichment p-value. The figures and table described below were rendered from STRING v.12.

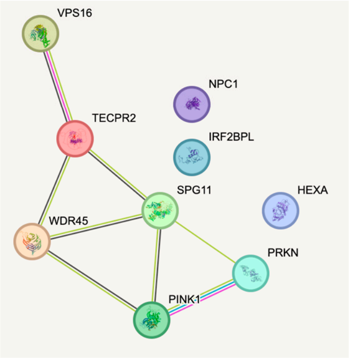

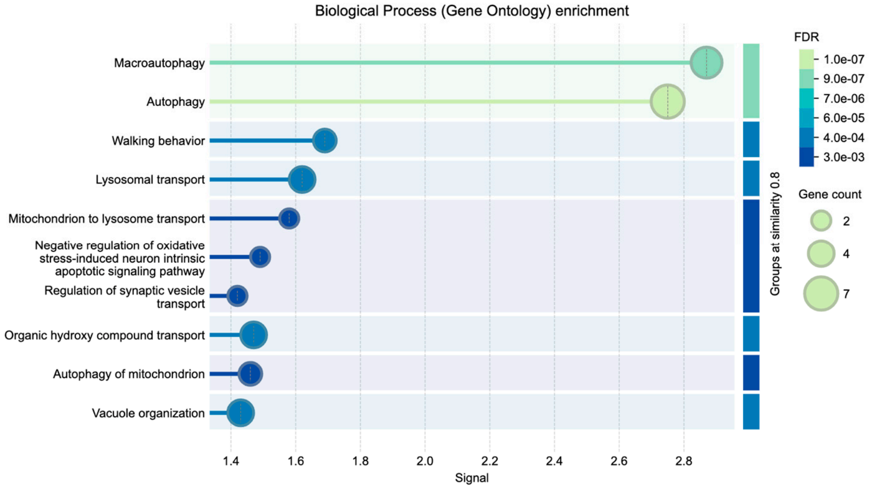

#Term ID	Term Description	Observed Gene Count	Background Gene Count	Strength	Signal	False Discovery Rate	Matching Proteins In Your Network (Ids)	Matching Proteins In Your Network (Labels)
PMID:34130600	(2022) The spectrum of neurodevelopmental, neuromuscular and neurodegenerative disorders due to defective autophagy.	6	93	2.15	2.75	5.73E-06	9606.ENSP00000261866,9606.ENSP00000269228,9606.ENSP00000348848,9606.ENSP00000352510,9606.ENSP00000355865,9606.ENSP00000364204	SPG11,NPC1,WDR45,TECPR2,PRKN,PINK1
PMID:34569973	(2022) A Practical Approach to Early-Onset Parkinsonism.	5	47	2.37	2.5	3.28E-05	9606.ENSP00000261866,9606.ENSP00000269228,9606.ENSP00000348848,9606.ENSP00000355865,9606.ENSP00000364204	SPG11,NPC1,WDR45,PRKN,PINK1

PMID:33931245	(2021) Moments in autophagy and disease: Past and present.	5	73	2.18	2.1	0.00014	9606.ENSP00000261866,9606.ENSP00000269228,9606.ENSP00000348848,9606.ENSP00000355865,9606.ENSP00000364204	SPG11,NPC1,WDR45,PRKN,PINK1
PMID:34308255	(2021) Towards a better understanding of the neuro-developmental role of autophagy in sickness and in health.	5	78	2.15	2.09	0.00014	9606.ENSP00000261866,9606.ENSP00000348848,9606.ENSP00000352510,9606.ENSP00000355865,9606.ENSP00000364204	SPG11,WDR45,TECPR2,PRKN,PINK1
PMID:32377374	(2020) Mendelian neurodegenerative disease genes involved in autophagy.	5	70	2.19	2.11	0.00014	9606.ENSP00000261866,9606.ENSP00000348848,9606.ENSP00000352510,9606.ENSP00000355865,9606.ENSP00000364204	SPG11,WDR45,TECPR2,PRKN,PINK1
PMID:23817214	(2013) The pallidopyramidal syndromes: nosology, aetiology and pathogenesis.	4	17	2.71	2.24	0.00014	9606.ENSP00000261866,9606.ENSP00000348848,9606.ENSP00000355865,9606.ENSP00000364204	SPG11,WDR45,PRKN,PINK1
PMID:36333361	(2022) A multicenter study of genetic testing for Parkinsons disease in the clinical setting.	4	32	2.44	1.76	0.00084	9606.ENSP00000261866,9606.ENSP00000348848,9606.ENSP00000355865,9606.ENSP00000364204	SPG11,WDR45,PRKN,PINK1
PMID:35856917	(2022) The apparent paradox of phenotypic diversity and shared mechanisms across dystonia syndromes.	4	34	2.41	1.73	0.00092	9606.ENSP00000238647,9606.ENSP00000269228,9606.ENSP00000348848,9606.ENSP00000369810	IRF2BPL,NPC1,WDR45,VPS16
PMID:33523105	(2021) Identification of Candidate Parkinson Disease Genes by Integrating Genome-Wide Association Study, Expression, and Epigenetic Data Sets.	4	35	2.4	1.73	0.00092	9606.ENSP00000261866,9606.ENSP00000348848,9606.ENSP00000355865,9606.ENSP00000364204	SPG11,WDR45,PRKN,PINK1
PMID:37304079	(2023) Genetic analysis of dystonia-related genes in Parkinsons disease.	4	45	2.29	1.52	0.0021	9606.ENSP00000238647,9606.ENSP00000355865,9606.ENSP00000364204,9606.ENSP00000369810	IRF2BPL,PRKN,PINK1,VPS16
PMID:32373609	(2020) MitophAging: Mitophagy in Aging and Disease.	4	45	2.29	1.52	0.0021	9606.ENSP00000269228,9606.ENSP00000348848,9606.ENSP00000355865,9606.ENSP00000364204	NPC1,WDR45,PRKN,PINK1
PMID:35505254	(2022) Macroautophagy in CNS health and disease.	4	50	2.24	1.46	0.0026	9606.ENSP00000261866,9606.ENSP00000348848,9606.ENSP00000355865,9606.ENSP00000364204	SPG11,WDR45,PRKN,PINK1
PMID:37906549	(2023) Levodopa-induced dyskinesia in early-onset Parkinsons disease (EOPD) associates with glucocerebrosidase mutation: A next-generation sequencing study in EOPD patients in Thailand.	4	53	2.22	1.44	0.0028	9606.ENSP00000261866,9606.ENSP00000348848,9606.ENSP00000355865,9606.ENSP00000364204	SPG11,WDR45,PRKN,PINK1
PMID:32145221	(2020) Impairment of Lysosome Function and Autophagy in Rare Neurodegenerative Diseases.	4	53	2.22	1.44	0.0028	9606.ENSP00000261866,9606.ENSP00000269228,9606.ENSP00000348848,9606.ENSP00000352510	SPG11,NPC1,WDR45,TECPR2
PMID:32472658	(2020) DNAJC6 Mutations Disrupt Dopamine Homeostasis in Juvenile Parkinsonism-Dystonia.	4	52	2.23	1.44	0.0028	9606.ENSP00000261866,9606.ENSP00000269228,9606.ENSP00000355865,9606.ENSP00000364204	SPG11,NPC1,PRKN,PINK1
PMID:26715604	(2016) Congenital disorders of autophagy: an emerging novel class of inborn errors of neuro-metabolism.	3	7	2.97	1.51	0.0028	9606.ENSP00000261866,9606.ENSP00000348848,9606.ENSP00000352510	SPG11,WDR45,TECPR2
PMID:31594250	(2020) Parkinsons Disease and Fabry Disease: Clinical, Biochemical and Neuroimaging Analysis of Three Pedigrees.	4	58	2.18	1.39	0.0033	9606.ENSP00000261866,9606.ENSP00000348848,9606.ENSP00000355865,9606.ENSP00000364204	SPG11,WDR45,PRKN,PINK1
PMID:25444595	(2015) NeuroX, a fast and efficient genotyping platform for investigation of neurodegenerative diseases.	4	59	2.17	1.39	0.0033	9606.ENSP00000261866,9606.ENSP00000269228,9606.ENSP00000355865,9606.ENSP00000364204	SPG11,NPC1,PRKN,PINK1
PMID:36468052	(2022) Case report: Early-onset Parkinsons disease with initial spastic paraparesis and hyperreflexia caused by compound heterozygous PRKN-gene exon 2 and 4 deletions.	3	11	2.78	1.29	0.0063	9606.ENSP00000261866,9606.ENSP00000355865,9606.ENSP00000364204	SPG11,PRKN,PINK1
PMID:20669327	(2010) Early-onset L-dopa-responsive parkinsonism with pyramidal signs due to ATP13A2,	3	11	2.78	1.29	0.0063	9606.ENSP00000261866,9606.ENSP00000355865,9606.ENSP00000364204	SPG11,PRKN,PINK1

	PLA2G6, FBXO7 and spatacsin mutations.							
PMID:37843206	(2024) Autophagy in neural stem cells and glia for brain health and diseases.	4	73	2.08	1.23	0.0064	9606.ENSP00000348848,9606.ENSP00000352510,9606.ENSP00000355865,9606.ENSP00000364204	WDR45,TECPR2,PRKN,PINK1
PMID:33662035	(2021) The CDT of Helicobacter hepaticus induces pro-survival autophagy and nucleoplasmic reticulum formation concentrating the RNA binding proteins UNRCSDE1 and P62SQSTM1.	4	75	2.07	1.21	0.0068	9606.ENSP00000269228,9606.ENSP00000348848,9606.ENSP00000355865,9606.ENSP00000364204	NPC1,WDR45,PRKN,PINK1
PMID:30369906	(2018) Parkinsonism in Inherited Metabolic Disorders: Key Considerations and Major Features.	3	12	2.74	1.27	0.0068	9606.ENSP00000269228,9606.ENSP00000355865,9606.ENSP00000364204	NPC1,PRKN,PINK1
PMID:20694531	(2010) Rare causes of dystonia parkinsonism.	3	13	2.7	1.24	0.0077	9606.ENSP00000261866,9606.ENSP00000355865,9606.ENSP00000364204	SPG11,PRKN,PINK1
PMID:36738194	(2023) Linking autism spectrum disorders and parkinsonism: clinical and genetic association.	3	14	2.67	1.2	0.0089	9606.ENSP00000348848,9606.ENSP00000355865,9606.ENSP00000364204	WDR45,PRKN,PINK1
PMID:17726045	(2007) In vitro and in silico analysis reveals an efficient algorithm to predict the splicing consequences of mutations at the 5 splice sites.	3	14	2.67	1.2	0.0089	9606.ENSP00000269228,9606.ENSP00000355865,9606.ENSP00000364204	NPC1,PRKN,PINK1
PMID:31299107	(2019) The genetic and clinico-pathological profile of early-onset progressive supranuclear palsy.	3	15	2.64	1.17	0.0099	9606.ENSP00000269228,9606.ENSP00000355865,9606.ENSP00000364204	NPC1,PRKN,PINK1
PMID:24386122	(2013) Niemann-Pick C disease gene mutations and age-related neurodegenerative disorders.	3	15	2.64	1.17	0.0099	9606.ENSP00000269228,9606.ENSP00000355865,9606.ENSP00000364204	NPC1,PRKN,PINK1
PMID:29945969	(2018) A Missense Mutation in the Vacuolar Protein Sorting 11 (VPS11) Gene Is Associated with Neuroaxonal Dystrophy in Rottweiler Dogs.	3	16	2.61	1.15	0.011	9606.ENSP00000348848,9606.ENSP00000352510,9606.ENSP00000369810	WDR45,TECPR2,VPS16
PMID:31450711	(2019) Mechanisms and Pathophysiological Roles of the ATG8 Conjugation Machinery.	4	93	1.97	1.08	0.0114	9606.ENSP00000348848,9606.ENSP00000352510,9606.ENSP00000355865,9606.ENSP00000364204	WDR45,TECPR2,PRKN,PINK1
PMID:33036336	(2020) Arylsulfatase A (ASA) in Parkinsons Disease: From Pathogenesis to Biomarker Potential.	3	17	2.59	1.12	0.0121	9606.ENSP00000269228,9606.ENSP00000355865,9606.ENSP00000364204	NPC1,PRKN,PINK1
PMID:30968111	(2019) A mutation in the major autophagy gene, WIPI2, associated with global developmental abnormalities.	3	18	2.56	1.09	0.0136	9606.ENSP00000348848,9606.ENSP00000355865,9606.ENSP00000364204	WDR45,PRKN,PINK1
PMID:33515275	(2021) Lipids, lysosomes and mitochondria: insights into Lewy body formation from rare monogenic disorders.	3	19	2.54	1.06	0.0153	9606.ENSP00000269228,9606.ENSP00000355865,9606.ENSP00000364204	NPC1,PRKN,PINK1
PMID:26555167	(2015) TECPR2 Associated Neuroaxonal Dystrophy in Spanish Water Dogs.	3	19	2.54	1.06	0.0153	9606.ENSP00000261866,9606.ENSP00000348848,9606.ENSP00000352510	SPG11,WDR45,TECPR2
PMID:35941989	(2022) Generalized dystonia without Parkinsonism in an LRRK2 carrier.	4	105	1.92	1	0.0156	9606.ENSP00000269228,9606.ENSP00000348848,9606.ENSP00000355865,9606.ENSP00000364204	NPC1,WDR45,PRKN,PINK1
PMID:34825060	(2021) A 3.9-Mb Deletion on 2p11.2 Comprising the REEP1 Gene Causes Early-Onset Atypical Parkinsonism.	3	20	2.52	1.05	0.0161	9606.ENSP00000261866,9606.ENSP00000355865,9606.ENSP00000364204	SPG11,PRKN,PINK1
PMID:26619007	(2015) Pompe disease: Shared and unshared features of lysosomal storage disorders.	3	20	2.52	1.05	0.0161	9606.ENSP00000269228,9606.ENSP00000355865,9606.ENSP00000364204	NPC1,PRKN,PINK1
PMID:34120768	(2021) Autophagy in liver diseases: A review.	4	109	1.9	0.98	0.0166	9606.ENSP00000269228,9606.ENSP00000348848,9606.ENSP00000355865,9606.ENSP00000364204	NPC1,WDR45,PRKN,PINK1

PMID:32894901	(2020) Young-Onset Parkinsons Disease with Impulse Control Disorder Due to Novel Variants of F-Box Only Protein 7.	3	22	2.47	1	0.0193	9606.ENSP00000261866,9606.ENSP00000355865,9606.ENSP00000364204	SPG11,PRKN,PINK1
PMID:35134347	(2022) The different autophagy degradation pathways and neurodegeneration.	4	115	1.88	0.94	0.0195	9606.ENSP00000261866,9606.ENSP00000348848,9606.ENSP00000355865,9606.ENSP00000364204	SPG11,WDR45,PRKN,PINK1
PMID:35845350	(2022) Autophagy in health and disease: From molecular mechanisms to therapeutic target.	4	116	1.88	0.94	0.0196	9606.ENSP00000348848,9606.ENSP00000355865,9606.ENSP00000364204,9606.ENSP00000369810	WDR45,PRKN,PINK1,VPS16
PMID:36611984	(2023) The Consequences of GBA Deficiency in the Autophagy-Lysosome System in Parkinsons Disease Associated with GBA.	3	24	2.44	0.99	0.0203	9606.ENSP00000269228,9606.ENSP00000355865,9606.ENSP00000364204	NPC1,PRKN,PINK1
PMID:37522749	(2023) The non-specific lethal complex regulates genes and pathways genetically linked to Parkinsons disease.	3	24	2.44	0.99	0.0203	9606.ENSP00000348848,9606.ENSP00000355865,9606.ENSP00000364204	WDR45,PRKN,PINK1
PMID:34375312	(2021) Identifying dominant-negative actions of a dopamine transporter variant in patients with parkinsonism and neuropsychiatric disease.	3	23	2.46	0.99	0.0203	9606.ENSP00000269228,9606.ENSP00000355865,9606.ENSP00000364204	NPC1,PRKN,PINK1
PMID:34654015	(2021) Levodopa-Induced Ocular Dyskinesia in an Early-Onset Parkinson Disease Patient With GBA Mutation.	3	25	2.42	0.99	0.**0203**	9606.ENSP00000261866,9606.ENSP00000355865,9606.ENSP00000364204	SPG11,PRKN,PINK1
PMID:32019516	(2020) Targeted next-generation sequencing identifies novel variants in candidate genes for Parkinsons disease in Black South African and Nigerian patients.	3	23	2.46	0.99	0.0203	9606.ENSP00000261866,9606.ENSP00000355865,9606.ENSP00000364204	SPG11,PRKN,PINK1
PMID:31248900	(2019) Sensory neuropathy and nociception in rodent models of Parkinsons disease.	3	23	2.46	0.99	0.0203	9606.ENSP00000269228,9606.ENSP00000355865,9606.ENSP00000364204	NPC1,PRKN,PINK1
PMID:31277291	(2019) A Comprehensive Review of Autophagy and Its Various Roles in Infectious, Non-Infectious, and Lifestyle Diseases: Current Knowledge and Prospects for Disease Prevention, Novel Drug Design, and Therapy.	4	121	1.86	0.93	0.0203	9606.ENSP00000261866,9606.ENSP00000348848,9606.ENSP00000352510,9606.ENSP00000364204	SPG11,WDR45,TECPR2,PINK1
PMID:30354903	(2018) Atg1-mediated autophagy suppresses tissue degeneration in pink1parkin mutants by promoting mitochondrial fission in Drosophila.	3	23	2.46	0.99	0.0203	9606.ENSP00000355865,9606.ENSP00000364204,9606.ENSP00000369810	PRKN,PINK1,VPS16
PMID:27913285	(2017) Autosomal recessive spinocerebellar ataxia 20: Report of a new patient and review of literature.	3	23	2.46	0.99	0.0203	9606.ENSP00000261866,9606.ENSP00000348848,9606.ENSP00000352510	SPG11,WDR45,TECPR2
PMID:24907664	(2014) Autophagy and human disease: emerging themes.	3	24	2.44	0.99	0.0203	9606.ENSP00000348848,9606.ENSP00000355865,9606.ENSP00000364204	WDR45,PRKN,PINK1
PMID:23462481	(2013) Monogenic Parkinsons disease and parkinsonism: clinical phenotypes and frequencies of known mutations.	3	24	2.44	0.99	0.0203	9606.ENSP00000261866,9606.ENSP00000355865,9606.ENSP00000364204	SPG11,PRKN,PINK1
PMID:22427796	(2012) Cooperative genome-wide analysis shows increased homozygosity in early onset Parkinsons disease.	3	23	2.46	0.99	0.0203	9606.ENSP00000261866,9606.ENSP00000355865,9606.ENSP00000364204	SPG11,PRKN,PINK1
PMID:34639106	(2021) Zebrafish and Medaka: Important Animal Models for Human Neurodegenerative Diseases.	3	26	2.4	0.96	0.0222	9606.ENSP00000269228,9606.ENSP00000355865,9606.ENSP00000364204	NPC1,PRKN,PINK1
PMID:30245141	(2018) The genetic landscape of Parkinsons disease.	3	26	2.4	0.96	0.0222	9606.ENSP00000261866,9606.ENSP00000355865,9606.ENSP00000364204	SPG11,PRKN,PINK1

PMID:35752680	(2022) Genetic landscape of early-onset dementia in Hungary.	3	27	2.39	0.94	0.0238	9606.ENSP00000261866,9606.ENSP00000355865,9606.ENSP00000364204	SPG11,PRKN,PINK1
PMID:22806825	(2012) The genetics and neuropathology of Parkinsons disease.	3	27	2.39	0.94	0.0238	9606.ENSP00000261866,9606.ENSP00000355865,9606.ENSP00000364204	SPG11,PRKN,PINK1
PMID:37935354	(2023) Structure and molecular basis of spermatid elongation in the Drosophila testis.	3	28	2.37	0.93	0.0254	9606.ENSP00000269228,9606.ENSP00000355865,9606.ENSP00000364204	NPC1,PRKN,PINK1
PMID:23804563	(2013) The Sac1 domain of SYNJ1 identified mutated in a family with early-onset progressive Parkinsonism with generalized seizures.	3	28	2.37	0.93	0.0254	9606.ENSP00000261866,9606.ENSP00000355865,9606.ENSP00000364204	SPG11,PRKN,PINK1
PMID:35409187	(2022) Novel Insights into Autophagy and Prostate Cancer: A Comprehensive Review.	3	29	2.35	0.91	0.0271	9606.ENSP00000269228,9606.ENSP00000355865,9606.ENSP00000364204	NPC1,PRKN,PINK1
PMID:35880381	(2022) Nearly Abolished Dopamine Transporter Uptake in a Patient With a Novel FBXO7 Mutation.	3	29	2.35	0.91	0.0271	9606.ENSP00000261866,9606.ENSP00000355865,9606.ENSP00000364204	SPG11,PRKN,PINK1
PMID:29881367	(2018) Frontotemporal Lobe Degeneration as Origin of Scans Without Evidence of Dopaminergic Deficit.	3	29	2.35	0.91	0.0271	9606.ENSP00000261866,9606.ENSP00000355865,9606.ENSP00000364204	SPG11,PRKN,PINK1
PMID:28042767	(2017) Advantages of Structure-Based Drug Design Approaches in Neurological Disorders.	3	29	2.35	0.91	0.0271	9606.ENSP00000269228,9606.ENSP00000355865,9606.ENSP00000364204	NPC1,PRKN,PINK1
PMID:28202669	(2017) The LRRK2-macroautophagy axis and its relevance to Parkinsons disease.	3	29	2.35	0.91	0.0271	9606.ENSP00000348848,9606.ENSP00000355865,9606.ENSP00000364204	WDR45,PRKN,PINK1
PMID:36073231	(2023) PTPA variants and impaired PP2A activity in early-onset parkinsonism with intellectual disability.	3	30	2.34	0.91	0.0275	9606.ENSP00000348848,9606.ENSP00000355865,9606.ENSP00000364204	WDR45,PRKN,PINK1
PMID:38137339	(2023) A Next-Generation Sequencing Study in a Cohort of Sicilian Patients with Parkinsons Disease.	3	30	2.34	0.91	0.0275	9606.ENSP00000261866,9606.ENSP00000355865,9606.ENSP00000364204	SPG11,PRKN,PINK1
PMID:35159316	(2022) Impact of Organelle Transport Deficits on Mitophagy and Autophagy in Niemann-Pick Disease Type C.	3	30	2.34	0.91	0.0275	9606.ENSP00000269228,9606.ENSP00000355865,9606.ENSP00000364204	NPC1,PRKN,PINK1
PMID:32498494	(2020) Long-Term Outcomes of Genetic Parkinsons Disease.	3	30	2.34	0.91	0.0275	9606.ENSP00000261866,9606.ENSP00000355865,9606.ENSP00000364204	SPG11,PRKN,PINK1
PMID:30917570	(2019) Analysis of 50 Neurodegenerative Genes in Clinically Diagnosed Early-Onset Alzheimers Disease.	3	30	2.34	0.91	0.0275	9606.ENSP00000261866,9606.ENSP00000355865,9606.ENSP00000364204	SPG11,PRKN,PINK1
PMID:33028204	(2020) Molecular evolutionary and structural analysis of human UCHL1 gene demonstrates the relevant role of intragenic epistasis in Parkinsons disease and other neurological disorders.	3	31	2.33	0.9	0.028	9606.ENSP00000261866,9606.ENSP00000355865,9606.ENSP00000364204	SPG11,PRKN,PINK1
PMID:31737044	(2019) The Role of Genetic Testing in the Clinical Practice and Research of Early-Onset Parkinsonian Disorders in a Hungarian Cohort: Increasing Challenge in Genetic Counselling, Improving Chances in Stratification for Clinical Trials.	3	31	2.33	0.9	0.028	9606.ENSP00000261866,9606.ENSP00000355865,9606.ENSP00000364204	SPG11,PRKN,PINK1
PMID:26413413	(2015) Separating from the pack: Molecular mechanisms of Drosophila spermatid individualization.	3	32	2.31	0.89	0.0298	9606.ENSP00000269228,9606.ENSP00000355865,9606.ENSP00000364204	NPC1,PRKN,PINK1

PMID:25253562	(2014) A random set scoring model for prioritization of disease candidate genes using protein complexes and data-mining of GeneRIF, OMIM and PubMed records.	3	33	2.3	0.87	0.032	9606.ENSP00000261866,9606.ENSP00000355865,9606.ENSP00000364204	SPG11,PRKN,PINK1
PMID:31991247	(2020) Genetics of Parkinsons disease: An introspection of its journey towards precision medicine.	3	34	2.29	0.85	0.0344	9606.ENSP00000269228,9606.ENSP00000355865,9606.ENSP00000364204	NPC1,PRKN,PINK1
PMID:28593578	(2017) Induced pluripotent stem cell-based modeling of neurodegenerative diseases: a focus on autophagy.	3	34	2.29	0.85	0.0344	9606.ENSP00000269228,9606.ENSP00000355865,9606.ENSP00000364204	NPC1,PRKN,PINK1
PMID:34360863	(2021) The Genetic Landscape of Parkinsonism-Related Dystonias and Atypical Parkinsonism-Related Syndromes.	3	35	2.27	0.83	0.0363	9606.ENSP00000269228,9606.ENSP00000355865,9606.ENSP00000364204	NPC1,PRKN,PINK1
PMID:37609280	(2023) The actin binding protein profilin 1 is critical for mitochondria function.	3	36	2.26	0.82	0.0388	9606.ENSP00000355865,9606.ENSP00000364204,9606.ENSP00000369810	PRKN,PINK1,VPS16
PMID:20950655	(2011) Mitochondrial involvement in cell death of non-mammalian eukaryotes.	3	36	2.26	0.82	0.0388	9606.ENSP00000269228,9606.ENSP00000355865,9606.ENSP00000364204	NPC1,PRKN,PINK1
PMID:28737703	(2017) Autophagy and Human Neurodegenerative Diseases-A Flys Perspective.	3	38	2.24	0.79	0.0441	9606.ENSP00000348848,9606.ENSP00000355865,9606.ENSP00000364204	WDR45,PRKN,PINK1
PMID:34768878	(2021) Targetable Pathways for Alleviating Mitochondrial Dysfunction in Neurodegeneration of Metabolic and Non-Metabolic Diseases.	3	39	2.23	0.77	0.0469	9606.ENSP00000269228,9606.ENSP00000355865,9606.ENSP00000364204	NPC1,PRKN,PINK1
PMID:30922179	(2019) Mitophagy and NAD(+) inhibit Alzheimer disease.	3	39	2.23	0.77	0.0469	9606.ENSP00000355865,9606.ENSP00000364204,9606.ENSP00000369810	PRKN,PINK1,VPS16
PMID:23132096	(2013) Membrane trafficking in neuronal maintenance and degeneration.	3	39	2.23	0.77	0.0469	9606.ENSP00000269228,9606.ENSP00000355865,9606.ENSP00000364204	NPC1,PRKN,PINK1
